# A cluster-control approach to a coronavirus disease 2019 (COVID-19) outbreak on a stroke ward with infection control considerations for dementia and vascular units

**DOI:** 10.1017/ice.2020.1437

**Published:** 2021-01-11

**Authors:** Emil P. Lesho, Edward E. Walsh, Jennifer Gutowski, Lisa Reno, Donna Newhart, Stephanie Yu, Jonathan Bress, Melissa Z. Bronstein

**Affiliations:** 1 Medicine Department, Rochester Regional Health, Rochester, New York; 2 Infectious Diseases Department, University of Rochester School of Medicine and Dentistry, Rochester, New York; 3 Quality and Safety Institute, Rochester Regional Health, Rochester, New York; 4 Surgery Department, Rochester Regional Health, Rochester, New York

**Keywords:** SARS-CoV-2, Outbreak Investigation, environmental contamination, patient behavior, PPE compliance

## Abstract

**Objective:**

We sought to contain a healthcare-associated coronavirus disease 2019 (COVID-19) outbreak, to evaluate contributory factors, and to prevent future outbreaks.

**Design:**

Quasi-experimental cluster-control outbreak evaluation.

**Methods:**

All patients and staff on the outbreak ward (case cluster), and randomly selected patients and staff on COVID-19 wards (positive control cluster) and a non-COVID-19 wards (negative control cluster) underwent reverse-transcriptase polymerase chain reaction (RT-PCR) testing. Hand hygiene and personal protective equipment (PPE) compliance, detection of environmental SARS-COV-2 RNA, patient behavior, and SARS-CoV-2 IgG antibody prevalence were assessed.

**Results:**

In total, 145 staff and 26 patients were exposed, resulting in 24 secondary cases. Also, 4 of 14 (29%) staff and 7 of 10 (70%) patients were asymptomatic or presymptomatic. There was no difference in mean cycle threshold between asymptomatic or presymptomatic versus symptomatic individuals. None of 32 randomly selected staff from the control wards tested positive. Environmental RNA detection levels were higher on the COVID-19 ward than on the negative control ward (OR, 19.98; 95% CI, 2.63–906.38; *P* < .001). RNA levels on the COVID-19 ward (where there were no outbreaks) and the outbreak ward were similar (OR, 2.38; *P* = .18). Mean monthly hand hygiene compliance, based on 20,146 observations (over preceding year), was lower on the outbreak ward (*P* < .006). Compared to both control wards, the proportion of staff with detectable antibodies was higher on the outbreak ward (OR, 3.78; 95% CI, 1.01–14.25; *P =* .008).

**Conclusion:**

Staff seroconversion was more likely during a short-term outbreak than from sustained duty on a COVID-19 ward. Environmental contamination and PPE use were similar on the outbreak and control wards. Patient noncompliance, decreased hand hygiene, and asymptomatic or presymptomatic transmission were more frequent on the outbreak ward.

Detailed descriptions of hospital-acquired coronavirus disease 2019 (COVID-19) and workplace transmission patterns are crucial to preventing outbreaks and improving patient safety.^1^ However, such descriptions in US hospitals remain scarce. These reports should be accompanied by an analysis of the specific infection control measures implemented.^1^ However, none of the major case series of COVID-19 patients from US hospitals, Italy, or China discuss infection control measures.^2–8^ Similarly, reports from skilled nursing facilities in the United States and London did not detail infection control measures beyond “restricting visitors, canceling communal events, and implementing COVID-19 transmission-based precautions.”^9–11^ Those reports did not explain those transmission-based precautions or evaluate other related factors such as compliance with personal protective equipment (PPE) and hand hygiene (HH), or the role of the environment in the outbreak.^9–11^An outbreak on a pediatric dialysis ward in Germany has also been described, but the details of the exposure are not provided. Other than saying that exposed staff were quarantined, specific infection control measures were not described.^12^

Although presymptomatic and asymptomatic transmission is increasingly recognized,^9,10,13–17^ until most recently, most COVID-19 testing in the United States has been performed on symptomatic individuals. This situation complicates nosocomial outbreak investigations because the prevalence of asymptomatic infections and the baseline seroprevalence are not fully known. However, this information is important for differentiating nosocomial transmission from background positivity rates and assessing the degree of spread throughout the ward.

## Objective

After the first nosocomial transmission of SARS-CoV-2 at our facility, we sought to contain the outbreak, to assess contributory factors, and to prevent future outbreaks using the information gained. In addition to those efforts, we describe the control measures implemented and their short-term impact.

## Outbreak

The outbreak occurred on a 39-bed acute-care stroke ward in a 528-bed teaching hospital. The layout of the ward and the sequence of secondary patient cases are illustrated in Supplementary Fig. 1 (online). Due to neurologic impairments in the patients, close physical contact with nursing and physical and occupational therapists occurs more frequently on this ward than on te general medical–surgical wards. Historically, nosocomial respiratory syncytial virus cases are more frequent on the ward compared to other wards. On average, 80% of the patients have intravenous catheters and 40% have indwelling or external urinary catheters.

The index case was a patient who had been on the stroke ward for 22 days before becoming acutely symptomatic with fever and lower respiratory tract symptoms. Her SARS-CoV-2 test was positive 20 hours later, and she required transfer to the intensive care unit (ICU) for endotracheal intubation. Prior to becoming symptomatic, the patient wandered throughout the ward visiting other patients and the nurse’s station without adhering to directions to wear a mask. To ameliorate nocturnal agitation that the patient suffered from, it was often necessary to place the patient in a geriatric chair at the nursing station. She was initially admitted for delirium and medication toxicity. During the admission, the patient also required continuous positive airway pressure and occasional bilevel positive airway pressure for chronic obstructive pulmonary disease.

**Fig. 1.**
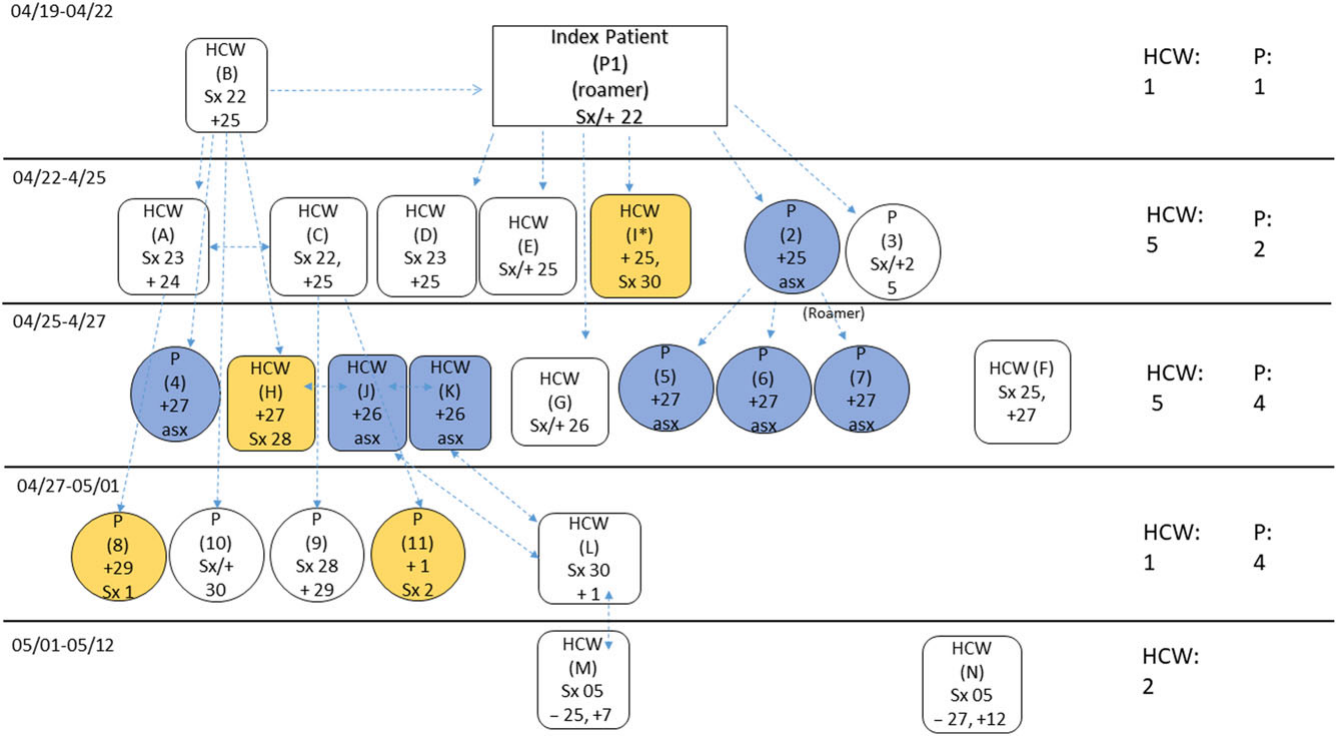
Transmission tree. Note. HCW, healthcare worker; Sx, symptomatic; asx, asymptomatic; —, presymptomatic transmission; (number), patient identifier; (letter), healthcare worker identifier; +, positive test; number, day of the month; yellow box/circle, presymptomatic; blue box/circle, asymptomatic.

One day after the index case became ill, a patient care technologist also became symptomatic and tested positive for SARS-CoV-2 the following day. All staff with exposure to the index case in the 14 days prior to her symptom onset, were offered SARS-CoV-2 screening. Three days after the index case was identified, a second patient on the ward, who was a frequent visitor of the index patient, also tested positive for SARS-CoV-2 (Supplementary Fig. 2 online). Both patients had a history of mental illness and were not compliant with requests to remain in their rooms or to use masks, despite repeated redirections from nursing staff. Subsequently, a second patient care technician became symptomatic and tested positive for SARS-CoV-2 (Fig. 1 and Supplementary Fig. 2 online).

## Methods

### Exposure definition

Due to the behavior of the index case, all patients on the outbreak ward and all staff who worked on that ward were considered exposed and included based on interim guidance.^18^ An exposed staff or patient was defined as a patient who was cared for by a SARS-CoV-2–positive staff member or who was within 2 m of a SARS-CoV-2–positive patient for at least 1 minute.

### Case definition

An individual case was defined as having a positive result for SARS-CoV-2 by real-time reverse transcription polymerase chain reaction (RT-PCR) (cobas 6800 System, Roche Diagnostics, Pleasanton, CA), or BD SARS-CoV-2 Reagents for BD MAX, (Becton Dickinson, Franklin Lakes, NJ). A hospital-acquired COVID-19 case was defined as a patient who had their first positive specimen collected after day 14 of their hospitalization.

### Containment and mitigation of outbreak

Prior to the outbreak, several COVID-19–specific infection control measures had already been adjusted to account for possible presymptomatic transmission. These included visitor restrictions, that is, no visitors allowed except in special circumstances, such as imminent death of the patient, and 1 birthing partner allowed. All such special circumstance visitors were interviewed in and questioned regarding symptoms and exposures, and their temperatures were measured. All healthcare workers were required to wear ASTM -Level 1 surgical masks. ASTM International, formerly known as American Society for Testing and Materials, is an international standards organization that develops and publishes technical standards. Staff who were positive for SARS-CoV-2 testing or symptomatic were restricted from working. SARS-CoV-2–positive patients were placed in cohorts on dedicated COVID-19 wards, and patients were required to wear masks when leaving their rooms. In addition to masks, all staff were provided face shields to wear when caring for COVID-19 or suspected COVID-19 patients. Face shields were required for COVID-19 patient care but were only recommended for non–COVID-19 patient care. Any patient having an aerosol-generating procedure was treated as a possible person under investigation. These aerosol-generating procedures required the use of N-95 filtering respirators and were performed in an airborne-infection isolation room whenever such a room was available.

Historically and currently, all wards were and are required to report monthly HH compliance to infection control and leadership committees. Clandestine observations are performed by specially trained staff (ie, ‘secret shoppers’). The same procedures apply to PPE observations.

Following the discovery of the index case, the entire outbreak ward was placed on quarantine precautions. As a result, gloves, face shield, and face mask were required for all patient contact on the ward (regardless of SARS-CoV-2 status) until 14 days after the last exposure. Patient assignments and work locations of staff were determined by interview and review of shift assignments. Quarantine information was given to the nurse manager and attending physicians from the ward to share with patients. Directly exposed patients (meaning patients who were visited by the index case or cared for by an employee who tested positive) were notified of their exposure and were placed in quarantine. Discharged COVID-19–exposed patients and their primary care physicians were notified by an ambulatory results nursing call center and were instructed to remain in home quarantine for the remainder of the 14 days following their last exposure. The list of exposed patients and staff was also reported to the local health department. New admissions to the stroke ward were restricted to patients with acute neurologic conditions.

Staff break-room capacity was limited through staggering and distanced seating. In addition to the routine regular monthly assessments, additional PPE and cleaning assessments were conducted clandestinely (by authors E.L., J.G., L.R., S.Y., and M.B). For an observation to be counted as compliant, the proper equipment had to be worn and worn correctly. For example, a cloth face mask covering the nose and mouth would not be considered compliant because staff were required to wear approved surgical masks. A surgical face mask covering the mouth but not the nose would also be considered noncompliant.

The same authors also observed patient behaviors regarding mask use on the outbreak and control wards. Additionally, safety and incident reports that involved behavior deviations by inpatients, and patient exposures, were reviewed.

### Management of exposed staff

Any employee who had spent time on the outbreak ward or had multiple interactions with patients and/or staff on that ward from 2 days before the index case became symptomatic to 7 days afterward was offered voluntary PCR testing for SARS-CoV-2. Staff were able to continue to work if asymptomatic while test results were pending (the universal masking policy was already in place). Staff who tested positive for SARS-CoV-2 were restricted from working until they were cleared by the employee health department (ie, at least 7 days following the test date, if remained asymptomatic, following Centers for Disease Control and Prevention [CDC] recommendations at the time). Staff who tested negative but had a defined medium- or high-risk exposure to a positive employee or patient were actively monitored for symptoms 14 days from exposure using the Datos Health Application (Ramat Gan, Israel).

### Selection of positive and negative control clusters/units

Before the outbreak occurred, the hospital had predesignated 1 of 4 ICUs and a general medicine floor as a COVID-19 unit in anticipation of the surge of COVID-19 patients. All patients testing positive for SARS-CoV-2 were admitted to this floor or ICU, and these were therefore selected as a positive control comparators. Two wards that had had no SARS-CoV-2–infected admission since the start of the pandemic, and that currently contained no such patients, were selected as the negative control comparators. These control wards and the outbreak ward are located on different floors. Patients and staff on the outbreak ward were considered the outbreak cluster.

### Asymptomatic testing

To assess the background point prevalence of asymptomatic SARS-CoV-2, every other patient across the negative-control comparator wards, and randomly selected staff on those wards were included and tested as ‘negative control clusters.’ Randomization was performed using a random number generator (1–26) corresponding to the first initial of the patient’s last name.

In response to the nosocomial case, all patients on the outbreak ward were tested for SARS-CoV-2. All new admissions to the outbreak ward were tested for COVID-19, upon or before arrival to the ward. Patients without typical or atypical COVID-19 symptoms were randomly selected as ‘negative control cluster’ from the non–COVID-19 wards. These wards also acted as the negative controls for serological and environmental assessments.

### Molecular testing, serology, environmental sampling

Nasopharyngeal specimens were analyzed on the cobas6800 System, (Roche Diagnostics), the BD MAX BioGX SARS-CoV-2 (Becton Dickinson), or the Simplexa COVID-19 Direct kit (DiaSorin Molecular, Minneapolis, MN).

Serology was performed 25–28 days after the index case was identified. Antibody binding to the SARS-CoV-2 spike and N proteins were detected in a standard 2-step enzyme immunosorbent assay using antibody-HRP-conjugate, goat anti-human IgG-antibody in an alkaline phosphatase substrate coated onto 96-well plates (Sigma-Aldrich, St Louis, MO). All serum was screened at 1:1,000 dilution, and all samples with an optical density 2-fold higher than the control well were confirmed as positive by 8 2-fold dilutions.

To ensure that the sampling method could reliably detect environmental RNA before formal sampling began, 100-cm^2^ test surfaces in vacated patient rooms were spotted with 1,250 copies of SARS-CoV-2 genomic RNA (BEI Resources). After 10 minutes of drying time, these surfaces were sampled as positive controls. All such controls tested positive using the commercial assay described below.

Environmental surface sampling was performed as previously described.^19,20^ Briefly, swabs were streaked over targeted high-touch surfaces for a minimum of 20 seconds in a rolling motion to ensure contact with the entire swab surface. The cobas PCR Media Uni swabs (Roche) were premoistened with the transport media (sterile 40% guanidine hydrochloride tris-HCl buffer) and analyzed on the cobas 6800 System (Roche).

Eight stationary surfaces were sampled in rooms on the outbreak and control wards: bed rails, call buttons and remote controls, over-bed tray tables, sinks and soap dispensers, chairs, windowsills, and floors. Three shared surfaces were sampled: walking computer workstations, handheld glucometers, and vital-sign machines. Staff break rooms, including microwaves and refrigerators, were also sampled.

The χ^2^ test, the Fisher exact test, and analysis of variance (ANOVA) tests were performed using R software (R Foundation for Statistical Computing, Vienna, Austria) or Minitab 2020 software (Minitab, State College, PA).

## Results

In total, 145 staff and 26 patients from the outbreak ward were potentially exposed. Overall, 11 staff declined testing, and 5 staff and 2 patients were not available for testing. Furthermore, 14 of 129 staff and 10 of 24 patients tested positive for SARS-CoV-2 (Table 1). Also, 4 of 14 (29%) positive staff were either asymptomatic (n = 2), or presymptomatic (n = 2). Of 10 patients, 7 (70 %) were either asymptomatic (n = 5) or presymptomatic (n = 2) at the time their initial PCR was positive (Fig. 1 and Supplementary Fig. 2 online). None of 32 randomly selected staff from the positive and negative control wards tested positive (Table 1).

**Table 1.** Outcome Measures on the Outbreak Ward Compared to the Positive and Negative Control Wards

Measurement	Outbreak Ward, No./Total (%)	Negative Control Ward, No./Total (%)	Positive Control Ward, No./Total (%)	*P* Value Outbreak vs Controls
**RT-PCR**				
Staff	14/129 (11%)	0/16 (0%)	0/16 (0%)	
Patients	10/24 (42%)	0/31(0%)	30/30 (100%)	
IgG antibody staff	9/29 (31%)	1/32 (3%)	6/79 (8%)	**.008** (OR, 3.78; 95% CI, 1.01–14.25)
**PPE compliance**				>.05
Mask	80/88 (91%)	87/106 (82%)	110/140 (79%)	
Gloves	10/13 (77%)	8/15 (53%)	32/38 (84%)	
Gowns	5/5 (100%)	2/2 (100%)	26/28 (93%)	
Mean monthly hand hygiene compliance	589/962	826/1130	2002/2534	**<.006**
	(61%)	(74%)	(79%)	
	N=962	N=1130	N=2534	
Environmental surface positivity RNA^a^	7/33	1/33	13/33	.18 (OR, 2.38; 95% CI, 0.72–8.47)

Note. PPE, personal protective equipment.

a Does not include 5 /12 dialysis machines and 0/14 other surfaces shared among wards.

In total, 140 staff from the control wards and outbreak ward volunteered for SARS-CoV-2 antibody testing using the cobas assay under an research protocol approved by the institutional review board. Of these, 126 were negative and 16 were positive. Compared to the control wards, the proportion of staff with detectable antibodies was higher on the outbreak ward (OR, 3.78; 95% CI, 1.01–14.25) (Table 1).

Of 128 randomly sampled surfaces on the outbreak and control wards, 26 were positive. Floors, call buttons and remote controls, chairs, and shared equipment (including glucometers and dialysis machines) were the surfaces most commonly positive for SARS-CoV-2 RNA. The proportion of tested surfaces that were positive for SARS-CoV-2 RNA was higher on the dedicated COVID-19 ward (positive control) than on the non–COVID-19 ward (negative control ward; OR, 19.98; 95% CI, 2.63–906.38; *P* < .001). However, there was no difference in the proportion of tested surfaces that were positive between the dedicated COVID-19 ward (where there had been no outbreaks) and the outbreak ward (OR, 2.38; 95% CI, 0.72–8.47; *P* = .18).

**Fig. 2.**
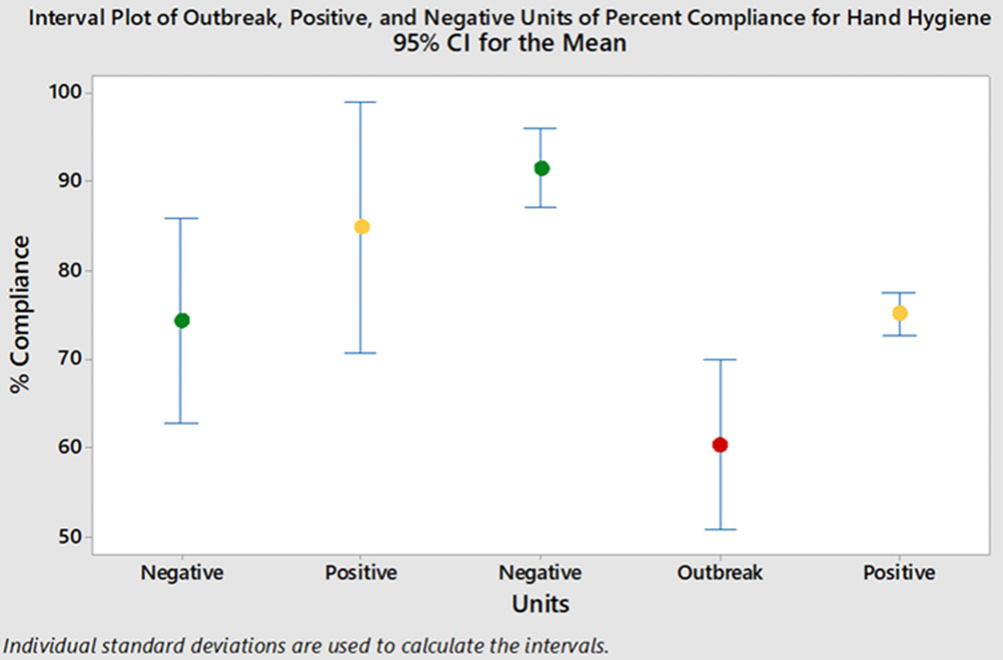
Mean monthly hand hygiene compliance. Note. Green, negative control wards; yellow, positive control wards; red, outbreak ward.

For the year prior to the outbreak, there were 20,146 clandestine HH observations (ie, ‘secret shopper’) across all wards at the hospital. The outbreak ward had lower mean monthly HH compliance than most other wards. Of the 20,146 observations, 4,626 were on the outbreak and control wards. Mean monthly HH compliance was significantly lower on the outbreak ward than the positive and negative control wards, averaging 61% on the outbreak ward compared to 79% and 74% on the positive and negative control wards, respectively (*P* ≤ .006) (Fig. 2).

There were 354 clandestine observations for compliance with COVID-19–specific PPE recommendations immediately after the identification of the index case. Overall PPE compliance was 91% on the outbreak ward versus 79% on the positive control ward and 82% on the negative control ward, but the differences were not significant (all *P* > .05). There were no patient safety or incident reports, and 0 of 72 patients were observed without a mask on the positive and negative control wards.

## Discussion

We found no other cluster control reports of a nosocomial outbreak in a US acute-care hospital that detailed specific control measures and also evaluated compliance with PPE recommendations, environmental surface contamination with SARS-CoV-2, and staff seroconversion. To our knowledge, this report appears to be the first such report. Furthermore, reports from non–university-based locations, such as this one, are underrepresented in research; most studies are carried out in large academic institutions. However, most health care in the United States is provided at non–university-based facilities. Additionally, the factors involved in this outbreak (requirement for aerosol generating procedures, uncontrolled behaviors, pre-symptomatic transmission) have been associated with explosive outbreaks and sustained transmission (super-spreading events).^21,22^ However, the secondary attack rate of 14% (24 of 171 exposures) in this report is lower than those in most available reports.^23–25^

The observation that infection in staff, as measured by seroconversion, was not higher on a dedicated COVID-19 ward compared to wards that had no patients with SARS-CoV-2 infection suggests that the prevailing PPE practices described here were effective. However, unanticipated movements or events are known to be factors in workplace accidents, and those appear to have furthered this outbreak. Similarly, decreased HH compliance likely contributed as well. Presymptomatic and asymptomatic transmission is increasingly recognized,^9,13–17^ and nearly all the transmission that occurred in this outbreak was either presymptomatic or asymptomatic (Supplementary Fig. 2 online).

This report has several limitations. Because of its quasi-experimental nature, causality cannot be inferred. It is from a single facility, but the facility is typical of many hospitals and settings in the United States, so the findings are still relatively generalizable. The number of PPE observations was unavoidably small given the limited number of trained observers and competing duties. We did not have the ability to cultivate SARS-CoV-2 virus, so another limitation is that the infectivity of the environmental RNA is unknown.

Our findings raise several unanswered questions that are important considerations for future control efforts. These issues include the legal, ethical, and safety implications of managing noncompliant, infectious, or potentially infectious patients. These challenges and questions have particular implication for neurobehavioral wards where a patient’s understanding and behavior are degraded or risky, and for other vascular wards where symptoms of SARS-CoV-2 infection can overlap with or be mistaken for underlying cardiac and vascular conditions such as stroke, cardiac insufficiency, thromboembolism, or even syncope (Table 2).^26^

**Table 2.** SARS-CoV-2 Infection Control Considerations for Stroke, Dementia, and Vascular Units

Unit-Specific Challenge	Consideration Before a Nosocomial Case(s)	Consideration Immediately After a Nosocomial Case(s)
Patients with plegias or paralysis require prolonged close contact for ADL	Require full PPE including face shield or goggles	Obtain leadership support and unit-level champions
COVID-19 can present as stroke, syncope,^26^ other cardiac events	Regularly assess PPE compliance	Conduct huddles with all involved staff
Memory impaired or dementia patients unable to comply with mask or movement restrictions	Place patients needing AGPs in private rooms if possible	Test all staff
Time-dependent false-negative rate increases possibility that initial admission test is false negative^27^	Test all patients upon admission, cohort positive patents	Retest all patients, and given false negative rate, a single repeat 2 or 3 days after admission, may not be enough^27^
Risk of staff exposure and seroconversion was significantly higher during a brief outbreak on stroke/vascular/dementia ward than during sustained duty on a dedicated COVID-19 unit	Use N95 for all AGP regardless of initial test result	Re-emphasize full PPE use especially face shields/goggles; if N95 not used for AGP, then implement
	Consider hard stops in medical record in admission evaluation for assessments of preadmission COVID-19 exposures	Re-emphasize hand hygiene and re-educate on protean manifestations of SARS-CoV-2 infection
		Consider breakroom or carpool spread

Note. PPE, personal protective equipment; AGP, aerosol-generating procedure; ADL, activities of daily living; test, nasal pharyngeal swab for PCR.

For example, stroke is a potential complication of COVID-19, even in people with only mild symptoms, and one of the stroke patients on the outbreak ward may have remained undetected and may have caused the outbreak in the first place. To help mitigate this possibility, we have launched a pilot study repeating PCR testing 2–3 days after admission (Table 2).

The actions taken by the staff described herein, along with the support of hospital and system leadership and the local health department, probably averted a much worse scenario. However, achieving sustained mask use by inpatients and PPE compliance for all patient encounters (meaning masks plus eye protection) remains challenging and merits further research aimed at creative solutions for increasing compliance. Going forward, we are evaluating the effectiveness of highly visible HH and PPE ‘ambassadors’ who roam the wards providing friendly reminders and answering questions. We are also evaluating the feasibility of using whole-genome sequencing at nonuniversity hospitals such as this one to enhance outbreak investigations.

## Supplementary material

For supplementary material accompanying this paper visit https://doi.org/10.1017/ice.2020.1437. click here to view supplementary material
