# Supplementary figures and images for: A cluster-control approach to a coronavirus disease 2019 (COVID-19) outbreak on a stroke ward with infection control considerations for dementia and vascular units
Source: Infect Control Hosp Epidemiol. 2021 Jan 11:1–7. doi: 10.1017/ice.2020.1437 (PMC7870916; doi:10.1017/ice.2020.1437)

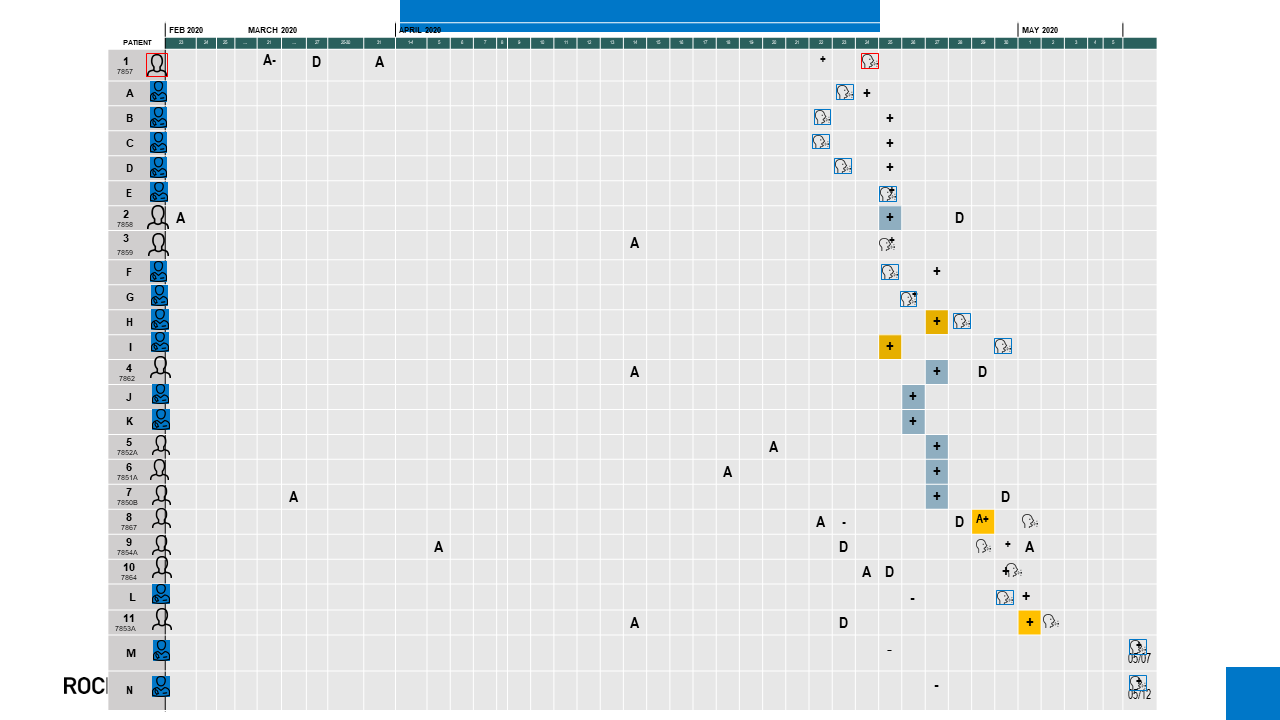

Supplement: Supplementary file 1 [file S0899823X20014373sup.zip › S0899823X20014373sup001.tif]

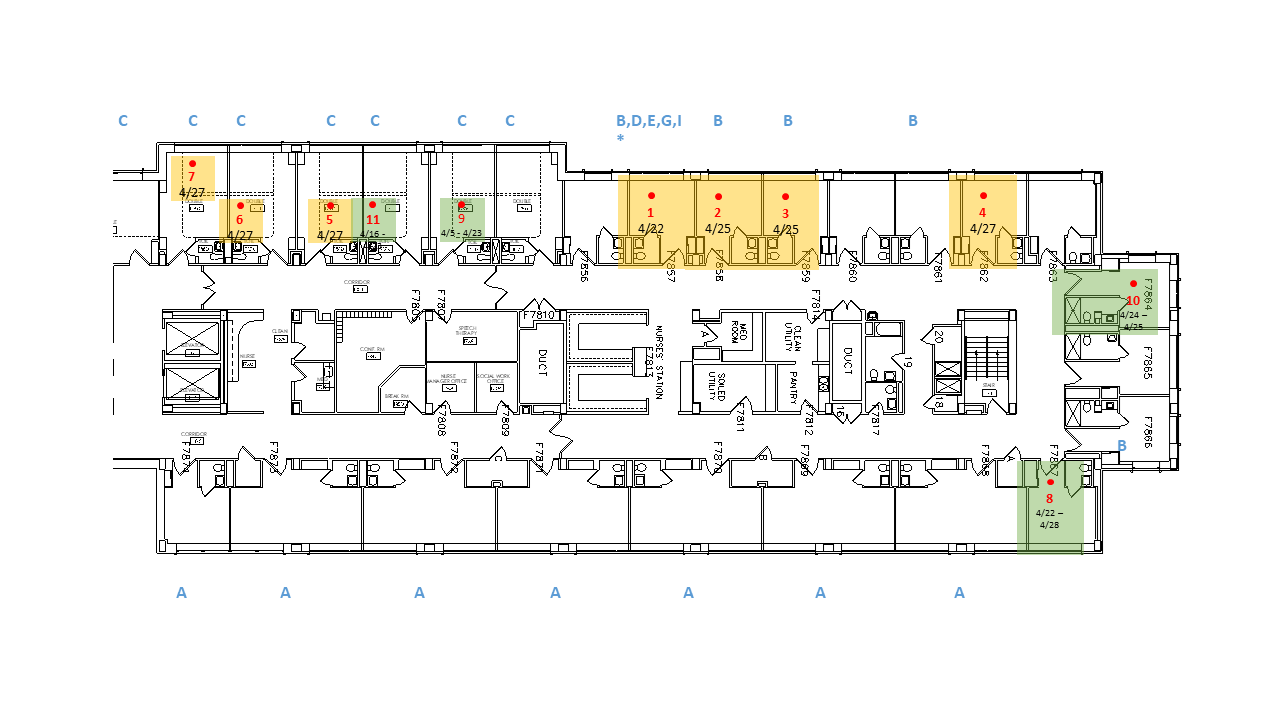

Supplement: Supplementary file 1 [file S0899823X20014373sup.zip › S0899823X20014373sup002.tif]
